# 基于代谢组学的多西他赛调控肺癌细胞代谢重编程研究

**DOI:** 10.3779/j.issn.1009-3419.2019.04.02

**Published:** 2019-04-20

**Authors:** 海超 孙, 海龙 朴, 欢 齐, 敏 颜, 宏旭 刘

**Affiliations:** 110042 沈阳，中国医科大学肿瘤医院胸外科 Department of Thoracic Surgery, Cancer Hospital, China Medical University, Shenyang 110042, China

**Keywords:** 多西他赛, 肺肿瘤, 气相色谱质谱联用, 代谢组学, 三羧酸循环, Docetaxel, Lung neoplasms, Gas chromatography-mass spectrometry, Metabolomics, Tricarboxylic acid cycle

## Abstract

**背景与目的:**

多西他赛是一种临床常用的抗肿瘤药物，是晚期非小细胞肺癌（non-small cell lung cancer, NSCLC）的一线用药。然而，多西他赛抗NSCLC作用的分子机制尚不明确。研究表明肿瘤细胞的代谢重编程在肿瘤发生发展过程中发挥重要作用。本研究旨在通过结合代谢组学分析及生物学手段来探讨多西他赛所影响的NSCLC细胞代谢通路。

**方法:**

首先，通过CCK-8实验分析多西他赛对NSCLC细胞活力的影响，筛选合适药物浓度。接下来，通过基于气相色谱质谱联用（gas chromatography-mass spectrometry, GC-MS）的代谢组学技术分析多西他赛处理和未处理的A549和H1299细胞。并通过统计学计算得到处理组和未处理组间的差异代谢物。最后，通过蛋白质免疫印迹分析（Western blot）多西他赛对其所调控的相关代谢途径中关键酶蛋白质表达水平的影响。

**结果:**

多西他赛可时间依赖和浓度依赖地抑制A549和H1299细胞活力。随着多西他赛处理时间延长，凋亡敏感蛋白质多聚二磷酸腺苷核糖聚合酶[Poly(ADP-)Polymerase, PARP]逐渐被激活裂解形成P89片段。代谢组学分析发现，药物处理后的A549和H1299细胞内，8种代谢物均发生显著变化，主要集中于三羧酸（tricarboxylic acid, TCA）循环代谢通路。同时，药物处理后，TCA循环关键调控酶异柠檬酸脱氢酶蛋白质表达水平显著下降。

**结论:**

多西他赛诱导NSCLC增殖抑制及凋亡的效应可能与下调异柠檬酸脱氢酶，进而抑制三羧酸循环代谢途径有关。

## Abstract (en)

**Background and objective:**

Docetaxel is a commonly used anti-tumor drug in clinic, especially as the first-line drug for advanced non-small cell lung cancer (NSCLC). However, the molecular mechanism of docetaxel against NSCLC is still unclear. Increasing studies have shown that metabolic reprogramming of tumor cells plays an important role in tumorigenesis. The aim of this study was to investigate the effects of docetaxel on the metabolic pathway of NSCLC cells based on metabolomics analysis and biological means.

**Methods:**

First, we performed CCK8 assay to analyze the effects of docetaxel on cell viability of NSCLC cells and also to screen the appropriate drug concentration. Then, the differential metabolites of docetaxel-treated and untreated NSCLC cells were analyzed by gas chromatography-mass spectrometry based metabolomics. Finally, the effects of docetaxel on the expression levels of key enzymes that regulate the relevant metabolic pathways were determined by Western blot.

**Results:**

Docetaxel inhibited cell viability of A549 and H1299 cells in a concentration- and time-dependent manner. With the prolonged treatment time of docetaxel, the apoptotic sensitive protein poly (ADP-ribose) polymerase (PARP) was gradually activated to form a P89 fragment. Metabolomics analysis showed that eight metabolites were significantly changed in both A549 and H1299 cells following docetaxel treatment, which were mainly in the tricarboxylic acid (TCA) cycle pathway. Moreover, after docetaxel treatment, the protein expression levels of isocitrate dehydrogenases, the key regulators of the TCA cycle, were obviously decreased in both A549 and H1299 cells.

**Conclusion:**

Our findings suggest that the effect of docetaxel-induced proliferation inhibition and apoptosis in NSCLC might be associated with down-regulation of isocitrate dehydrogenases and suppression of the TCA cycle pathway.

肺癌不仅是中国发病率和死亡率最高的恶性肿瘤，也是全世界范围内病死率最高的恶性肿瘤^[1]^，其中非小细胞肺癌（non-small cell lung cancer, NSCLC）患者约占肺癌患者总数的80%^[2]^。40%-50%的NSCLC患者在确诊时已经处于局部晚期或发生转移，5年生存率仅为5%。多西他赛（Docetaxel，多西紫杉醇）属于紫杉醇类，是一种微管稳定剂^[3]^，通过稳定微管蛋白质，抑制微管解聚和肿瘤细胞有丝分裂，发挥其抗肿瘤作用，临床上主要用于治疗NSCLC、乳腺癌、前列腺癌和宫颈癌等^[4]^。目前，多西他赛是治疗局部晚期或发生转移的NSCLC患者的一线用药^[5]^，其对顺铂治疗产生耐药性的患者也有效^[6]^。但是，多西他赛治疗NSCLC的分子机制尚未完全阐明。

代谢物变化是对细胞所进行生理活动的最直观反应，代谢物对研究细胞生理和病理机制具有重要意义，目前已鉴定出的生物体内代谢物大于4万个^[7]^。代谢组学是一种新兴的研究方法，是对整个生物体系总的代谢物所进行的全面定性定量研究^[8]^。其和基因组学、转录组学、蛋白质组学一起，成为系统生物学的重要研究方法。色谱-质谱联用以其高分辨力、高通量和高灵敏度的优势，已成为代谢组学的主流研究技术之一。基于气相色谱-质谱联用（gas chromatography-mass spectrometer, GC-MS）技术的代谢组学方法在检测糖酵解、三羧酸（tricarboxylic acid, TCA）循环、氨基酸和有机酸等代谢物中具有优势。并且，相对于其他代谢组学技术，GC-MS技术较成熟、仪器更加稳定、价格便宜^[9]^。

1922年瓦博格首次发现，肿瘤的发生发展与糖酵解代谢异常密切相关。在氧气充足的条件下，细胞大量摄取葡萄糖生成乳酸，激活相关信号通路和改变肿瘤微环境。相关研究表明，许多调控代谢的相关蛋白质可以调控细胞凋亡的发生，同时某些调控细胞凋亡的蛋白质也在代谢通路中发挥信使作用。靶向肿瘤代谢治疗的研究是基于代谢组学的方法，通过分析肿瘤异常代谢物和代谢通路，使用现有的生物化学技术合成新的靶向代谢的药物，为治疗肿瘤寻找新的突破点，为预防肿瘤寻找新的策略^[10]^。靶向肿瘤代谢的基本研究方法包括：①靶向代谢突变基因和激活的代谢基因；②回补肿瘤内缺失的代谢基因；③靶向代谢通路重编程等。

本文通过多西他赛处理NSCLC细胞后，使用基于GC-MS的代谢组学技术分析代谢物变化，并结合CCK-8和Western blot实验，对多西他赛影响NSCLC细胞A549和H1299的代谢物和代谢通路进行深入研究，从肿瘤代谢的角度解释多西他赛治疗NSCLC的作用方式。

## 1 材料与方法

### 1.1 仪器与试剂

使用GCMS-QP 2010Plus系统进行GC-MS代谢组学分析（日本岛津公司）。自动进样器：AOC-20i autosampler（日本岛津公司）。观察细胞使用倒置显微镜（德国Leica公司）。Western blot分析使用Fusion Fx化学发光仪（法国VILBER公司）。CO_2_培养箱、96孔板、细胞培养皿等均购自美国Thermo Fisher Scientific公司。

超纯水来自于Milli-Q水纯化系统（美国Millipore公司）。40 mg/mL多西他赛（溶于吐温80溶剂）购于齐鲁制药有限公司。甲氧胺盐酸盐、吡啶、二氯甲烷及N-甲基-N-（三甲基硅基）三氟乙酰胺（MSTFA）和十三酸均购自美国Sigma-Aldrich公司。色谱纯甲醇购自德国Merck公司。RPMI-1640培养基、胎牛血清（fetal bovine serum, FBS）、青霉素链霉素液（penicillin-streptomycin, PS）、磷酸缓冲盐溶液（phosphate buffer saline, PBS）、通用细胞冻存液和0.25%EDTA胰蛋白酶均购自美国Gibco公司。

人源NSCLC细胞A549和H1299购自中国科学院上海细胞库，并使用含10%FBS及1%PS的RPMI-1640培养基进行培养。细胞培养在5%CO_2_、37 ℃恒温孵箱中。细胞培养至密度为80%-90%时进行传代。

### 1.2 CCK-8测定细胞活力

活细胞内线粒体脱氢酶可与细胞增殖及毒性检测试剂（cell counting kit-8, CCK-8）反应变橙色。使用96孔板培养细胞，消化处于对数生长期的细胞，96孔板每孔接种3, 000-5, 000个细胞，24 h后A549和H1299细胞按照梯度0.3 nM、1 nM、3 nM、10 nM、30 nM、100 nM、300 nM、1, 000 nM处理多西他赛，平行设置三个对照组。继续培养24 h、48 h、72 h后，吸取上清后每孔按照1:9（CCK-8:培养基）加入CCK-8试剂100 μL，培养2 h后使用酶标仪（450 nm波长）测取并计算平均吸光度值A，细胞活力（%）=（A_处理组_-A_空白组_）/（A_对照组_-A_空白组_）。

### 1.3 蛋白质免疫印迹分析

分别取对数生长期的A549和H1299细胞2×10^6^个接种于4个10 cm^2^细胞培养皿。24 h后分别在培养基中加入30 nM和100 nM的多西他赛，处理0 h、6 h、12 h和24 h。弃去培养基，PBS清洗3次后。加入RIPA裂解液，刮下细胞，并将含细胞碎片的裂解液移至新离心管中。冰上裂解细胞1 h，每10 min涡旋一次。4 ℃，13, 000 g离心15 min，取上清。通过BCA法测定蛋白质浓度，等量蛋白质加入蛋白质上样缓冲液后，97 ℃变性10 min。通过SDS-聚丙烯酰胺凝胶电泳分离不同分子量大小的蛋白质，再将蛋白质转移至PVDF膜上。室温封闭1 h，4 ℃孵育一抗过夜。室温孵育二抗1 h，加入显影剂，化学发光成像仪检测蛋白质条带。本研究所用一抗：Vinculin，购自美国Sigma-Aldrich公司，1:1, 000稀释使用；PARP、IDH1和IDH2均购自美国Proteintech公司，1:1000稀释使用。

### 1.4 样品提取及衍生

分别取对数生长期的A549和H1299细胞2×10^6^个接种于10个10 cm^2^细胞培养皿。24 h后，5组作为处理组分别加入药物多西他赛30 nM（A549）或100 nM（H1299），5组作为对照组仅换液。药物处理24 h后，弃去培养基，使用5 mL冷PBS缓冲液轻柔冲洗细胞两次，立即液氮淬灭。加入含10 μg/mL十三酸的甲醇：水（4:1）1 mL，刮下细胞至（Eppendorf, EP）管中。涡旋两次，每次1 min。4 ℃，13, 000 *g*离心15 min，取上清，真空干燥冻干机（美国Labconco公司）中冻干^[11]^。

取出冻干样品，每个样品加50 μL甲氧吡啶（20 mg/mL），涡旋60 s，37 ℃水浴肟化1.5 h。再加入40 μL MSTFA，涡旋30 s，37 ℃水浴，硅烷化1 h。4 ℃，13, 000 *g*离心15 min。弃去不溶颗粒沉淀，取上清行GC-MS分析^[12]^。

### 1.5 GC-MS条件

色谱条件：使用DB-5 MS（30 μm×250 μm×0.25 μm；美国J & W Scientific公司）毛细管柱；使用程序性升温进行分析，起始温度为70 ℃，保持3 min后，以5 ℃/min速率升高温度至300 ℃，保持10 min；接口温度230 ℃；电离模式70 eV；进样量1 μL；载气线性速度40.0 cm/s，恒流模式流速1.19 mL/min；采用全扫描模式；扫描范围33 m/z-600 m/z。

### 1.6 数据处理与统计分析方法

GC-MS所获取的原始质谱数据（CDF格式）通过ChromaTOF 4.43软件（美国LECO公司）进行处理，结合数据库及组内标样，进行代谢物定性。通过GC-MS solution软件进行代谢物积分定量。得到代谢物峰表后，使用SIMCA-P 11.0软件（瑞典Umetrics公司）进行偏最小二乘法判别分析，绘制PLS-DA得分图，可视化组间聚集及离散程度。运用统计学*t*检验方法（*P* < 0.05），得到对照组和多西他赛处理组的A549和H1299细胞差异代谢物。使用MeV热图软件绘制差异代谢物热图，使用MetaboAnalyst 3.0在线软件进行通路富集分析，使用GraphPad Prism 5绘图软件制作柱状图。

## 2 结果

### 2.1 多西他赛对NSCLC细胞A549和H1299的抑制作用

为探讨多西他赛对NSCLC增殖的影响，本实验采用CCK-8试剂盒测定了多西他赛对A549和H1299细胞活力的作用。图 1A-图 1B为多西他赛8个浓度梯度和3个作用时间对A549和H1299细胞活力的影响。随着多西他赛药物浓度升高和作用时间延长，A549和H1299细胞活力下降，多西他赛对NSCLC的增殖抑制作用具有明显的浓度和时间依赖性。

**1:**
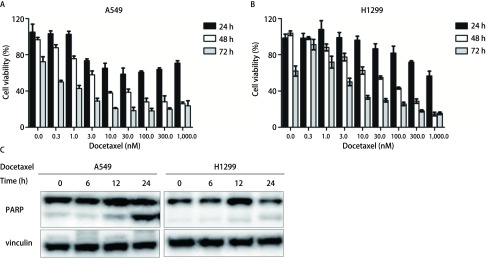
不同浓度和时间处理多西他赛对A549和H1299细胞活力和凋亡的影响。A-B：不同多西他赛浓度（0.3 nM、1 nM、3 nM、10 nM、30 nM、100 nM、300 nM、1, 000 nM）及作用时间（24 h、48 h、72 h）对A549（A）和H1299（B）细胞活力的影响；C：多西他赛处理A549（浓度30 nM）和H1299（浓度100 nM）细胞（处理时间0 h、6 h、12 h、24 h）后，凋亡蛋白质PARP表达量变化。Vinculin作为内参蛋白质。 Effects of docetaxel with different concentrations and reaction times on the cell viability and apoptosis in A549 and H1299 cells. A-B: Effects of docetaxel with different concentrations (0.3 nM, 1 nM, 3 nM, 10 nM, 30 nM, 100 nM, 300 nM, 1, 000 nM) and reaction times (24 h, 48 h and 72 h) on the cell viability and apoptosis in A549 (A) and H1299 (B) cells; C: The expression of poly ADP-ribose polymerase (PARP) in A549 (30 nM) and H1299 (100 nM) cells after docetaxel treatment for 0 h, 6 h, 12 h and 24 h. Vinculin was used as a loading control.

为探讨多西他赛对NSCLC凋亡的作用。通过Western blot实验，测定多西他赛作用A549和H1299细胞0 h、6 h、12 h和24 h时，凋亡敏感指标PARP蛋白质表达。结果表明，随着多西他赛处理时间延长，A549和H1299细胞中的PARP蛋白质均逐渐被激活裂解形成P89片段（图 1C），细胞发生凋亡。上述结果提示多西他赛能诱导NSCLC发生凋亡。

### 2.2 GC-MS代谢组学技术分析多西他赛处理前后的A549和H1299细胞差异代谢物

基于代谢组学中需要活细胞提取代谢物进行分析的基础上，综合考虑多西他赛对NSCLC增殖和凋亡的影响，保证多西他赛的药理作用，同时保持相当程度的细胞活力。根据CCK-8实验结果，选择作用A549细胞的药物浓度为30 nM，作用H1299细胞的药物浓度为100 nM，作用时间为24 h，A549和H1299细胞活力均在70%-80%之间，实验具有可行性。

提取代谢物前使用液氮淬灭细胞，去除微环境变化对细胞代谢状态的影响^[14]^。依前述方法样品经提取硅烷化衍生，利用GC-MS技术分析细胞代谢物。得到对照组和多西他赛处理组A549和H1299细胞代谢物的GC-MS总离子流图。

考虑到非药物因素的影响，排除复杂的环境条件干扰，最大化组间分离，并寻找各组间的差异代谢物。进一步通过偏最小二乘法判别分析（partial least square discriminant analysis, PLS-DA）经行多元数据处理。图 2是对照组和处理组A549和H1299细胞代谢物全谱数据的PLS-DA得分图，该模型包含两个主成分^[14]^，其中R^2^X=0.831、R^2^Y=0.983和Q^2^=0.878，提示该模型具有较高的稳定性和较好的预测率。在PLS-DA得分图上，对照组和处理组A549和H1299细胞代谢物可以明显的区分开。

**2:**
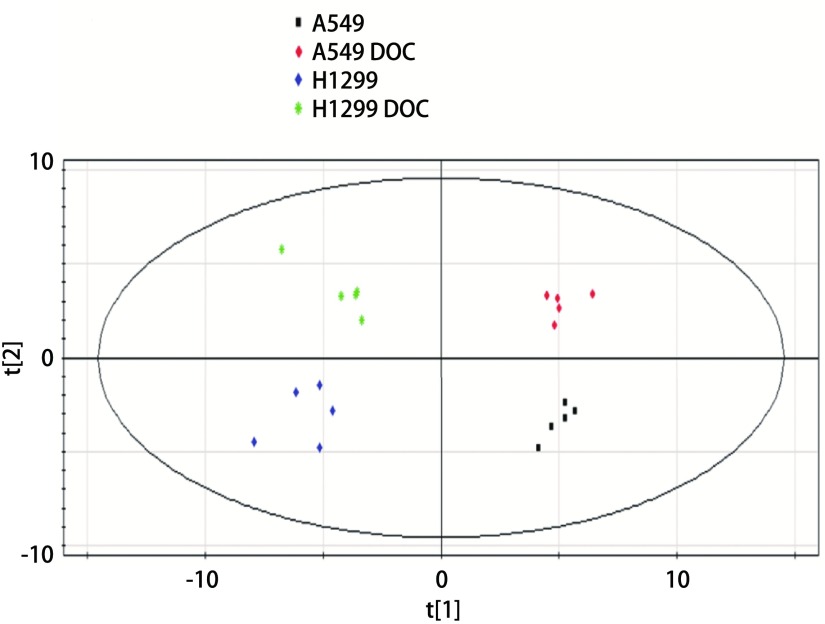
对照组和多西他赛处理24 h的A549和H1299细胞代谢物的PLS-DA得分图（*n*=5） PLS-DA score plot of control and docetaxel treated A549 and H1299 cells for 24 h (*n*=5)

结合*t*检验的统计方法，得到多西他赛处理前后的A549差异代谢物31种和H1299细胞差异代谢物19种，MeV热图分析提示处理组的大部分代谢物含量下降(图 3A-图 3B）。处理组A549细胞的丙酮酸、丙酸、甘氨酸等25种代谢物含量下降，丝氨酸、磷酸、腺嘌呤等6种代谢物含量上升。处理组H1299细胞的乳酸、琥珀酸、胆固醇等11种代谢物含量下降，乳糖、木糖醇、葡萄糖等8种代谢物含量上升。

**3:**
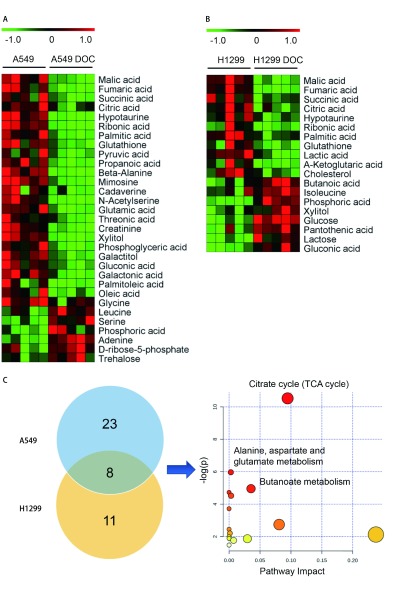
多西他赛对A549和H1299细胞中代谢物和代谢通路的影响（*n*=5）。A-B：对照组和多西他赛处理24 h组的A549细胞（A）和H1299细胞（B）差异代谢物的热图；C：韦恩图统计A549和H1299细胞中共同的差异代谢物，并对8种差异代谢物进行通路富集分析。 Effects of docetaxel on metabolites and metabolic pathways in A549 and H1299 cells (*n*=5). A-B: Heatmap of metabolites in A549 cells (A) and H1299 cells (B) following docetaxel treatment for 24 h; C: Venn diagram of commonly differential metabolites in both A549 and H1299 cells. Pathway Impact analysis results by 8 differential metabolites.

### 2.3 多西他赛可下调NSCLC的TCA循环代谢途径

多西他赛处理组A549和H1299的共同差异代谢物包括：苹果酸（malic acid）、富马酸（fumaric acid）、琥珀酸（succinic acid）、柠檬酸（citric acid）、亚牛磺酸（hypotaurine）、核糖酸（ribonic acid）、棕榈酸（palmitic acid）和谷胱甘肽（glutathione），在药物处理后含量下降。通过使用在线工具MetaboAnalyst 3.0软件，进行8种共同差异代谢物的通路富集分析（图 3C），结果显示主要影响TCA循环代谢途径（*P*=0.002, 14）。

图 4A-图 4E所示为多西他赛处理后含量下降的TCA循环中间产物。苹果酸、富马酸、琥珀酸、柠檬酸在处理组的A549细胞中含量下降。苹果酸、富马酸、琥珀酸、柠檬酸、α-酮戊二酸（α-ketoglutaric acid）在处理组的H1299细胞中含量下降。

**4:**
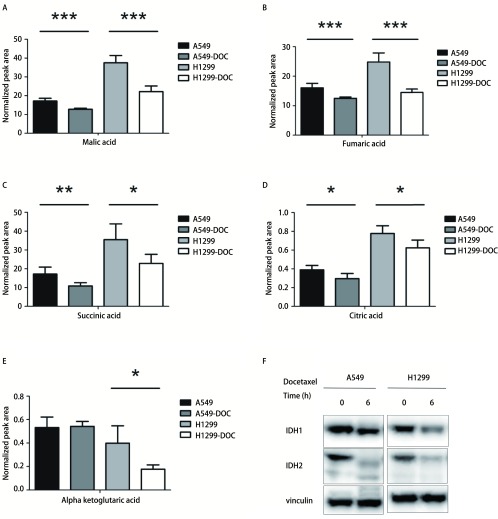
多西他赛对A549和H1299细胞的TCA循环的影响。A-E：对照组和多西他赛处理24 h的A549和H1299细胞中TCA循环中间产物：苹果酸、富马酸、琥珀酸、柠檬酸、α-酮戊二酸变化情况；F：对照组和多西他赛处理6 h的A549和H1299细胞的TCA循环关键酶IDH1和IDH2蛋白质的表达。*n*=5，*P < 0.05，**P < 0.01及*** *P* < 0.001。Vinculin作为内参蛋白质。 Effects of docetaxel on TCA cycle in A549 and H1299 cells. A-E: Alterations of intermediate metabolites of TCA cycle, included malic acid, fumaric acid, succinic acid, citric acid, α-ketoglutaric acid, in control and docetaxel treatment group in A549 and H1299 cells for 24 h; F: The expression of the key enzymes protein of TCA cycle, included isocitrate dehydrogenase 1 and isocitrate dehydrogenase 2, in control and docetaxel treatment group in A549 and H1299 cells for 6 h. *n*=5, *P < 0.05, **P < 0.01, ***P < 0.001 *vs* the control group. Vinculin was used as a loading control.

异柠檬酸脱氢酶（isocitrate dehydrogenase, IDH）是TCA循环过程中的关键酶，可催化异柠檬酸转化为α-酮戊二酸。对对照组和多西他赛处理6 h后的A549和H1299细胞进行Western blot分析，药物处理6 h后IDH1和IDH2蛋白质的表达量下降（图 4F）。推测多西他赛通过抑制TCA循环关键酶，下调NSCLC细胞的TCA循环。

## 3 讨论

本研究通过CCK-8实验、Western blot分析，使用基于GC-MS技术的代谢组学方法，结合多种统计软件，探索多西他赛对A549和H1299细胞代谢的影响。结果表明，多西他赛对NSCLC的主要作用有：①可浓度和时间依赖地抑制细胞活力和诱导细胞凋亡；②下调TCA循环的中间代谢物：苹果酸、富马酸、琥珀酸和柠檬酸含量，同时也下调亚牛磺酸、核糖酸、棕榈酸、谷胱甘肽的含量；③下调TCA循环的关键酶异柠檬酸脱氢酶蛋白质水平。

TCA循环是产生ATP的一条重要电子传递链，主要在生物体细胞的线粒体内进行^[15]^，是生物能量的重要来源，同时也为肿瘤细胞增殖提供了必要条件。TCA循环与NSCLC的发生发展密切相关^[16]^。TCA循环中的关键酶：琥珀酸脱氢酶（succinate dehydrogenase）、异柠檬酸脱氢酶和延胡索酸水合酶（fumarate hydratase）突变表达的非小细胞肺癌患者有着更高的复发率和死亡率^[17]^。研究^[18]^表明，通过分析注射同位素^13^C标记葡萄糖肺癌患者的肺组织，相比正常肺组织，肺癌组织内大量的^13^C富集在琥珀酸和柠檬酸中。大量葡萄糖经过TCA循环转化为脂质、蛋白质和核酸以满足肺癌组织生长的高合成代谢需求。

靶向代谢重编程药物的研究，主要是针对肿瘤细胞内存在的异常代谢。瓦博格提出，在氧气绝对充足的条件下，相比于正常组织，肿瘤组织的细胞内氧化磷酸化被抑制^[19]^，糖酵解水平升高200倍以上（瓦博格效应）。糖酵解通过转化为谷氨酰胺合成α-酮戊二酸，参与TCA循环，促进肿瘤的进一步发展^[20]^。添加外源柠檬酸，负反馈调节抑制肿瘤细胞的TCA循环可抑制A549细胞的增殖和裸鼠异体移植瘤的生长^[21]^，研究结果表明对代谢重编程的调控可以治疗NSCLC。

代谢组学是研究生物体系内各种生理活动调节后最终状态的学科，为本研究提供了重要的理论基础。GC-MS气相色谱质谱联用技术，为本研究提供了技术上的可行性。通过对差异代谢物的代谢组学分析，发现多西他赛作用于NSCLC细胞后TCA循环等代谢通路的改变，证明了多西他赛对代谢通路调控作用。后续实验需进一步验证多西他赛作用于TCA循环的机制，三羧酸循环影响NSCLC的机制和多西他赛对代谢调控作用的动物实验。希望可通过本研究，为靶向代谢重编程药物的开发和药理学研究提供一种新的策略。
